# OLDER ADULT PERSPECTIVES ABOUT ONLINE EXERCISE CLASSES DURING THE COVID-19 PANDEMIC AT MULTIPLE TIME POINTS

**DOI:** 10.1093/geroni/igac059.2608

**Published:** 2022-12-20

**Authors:** Michelle Porter, Dallas Murphy, Nicole Dunn, Ruth Barclay, Stephen Cornish, Jacquie Ripat, Kathryn Sibley, Sandra Webber

**Affiliations:** University of Manitoba, Winnipeg, Manitoba, Canada; University of Manitoba, Winnipeg, Manitoba, Canada; University of Manitoba, Winnipeg, Manitoba, Canada; University of Manitoba, Winnipeg, Manitoba, Canada; University of Manitoba, Winnipeg, Manitoba, Canada; University of Manitoba, Winnipeg, Manitoba, Canada; University of Manitoba, Winnipeg, Manitoba, Canada; University of Manitoba, Winnipeg, Manitoba, Canada

## Abstract

Engaging in physical activity can bring health benefits for older adults. However, during the pandemic the availability of in-person exercise classes has been sporadic. As such, online exercise programs have become more common. This research had the goal of exploring the uptake of online exercise programs by older adults in Manitoba, Canada in the first few months in the pandemic and then more than 1.5 years into the pandemic. Older adults (65 years and older) were recruited via emails from a variety of community organizations. Participants completed anonymous online surveys in summer 2020 (n=678) and fall 2021 (n=570). Less than 50% of respondents reported participating in online exercise classes during the pandemic in both surveys. For both surveys, pre-recorded classes were the most common, however, this decreased from 80% in the first survey, to 57% in the second survey. Conversely, live classes where the instructor could see the participants increased from 17% in the 2020 survey, to 47% in the 2021 survey. Additionally, platform use shifted from YouTube as the most popular in the first survey, to Zoom in the second survey. Most of the online classes originated from their local communities. Of those who participated in online exercise early and later in the pandemic, about two thirds reported that they would continue online exercise classes outside of the pandemic. A major reason for not participating was because they enjoy the social aspect of in-person classes. The perspectives of the study participants will be valuable for policymakers, programmers, and instructors.

